# TREE2FASTA: a flexible Perl script for batch extraction of FASTA sequences from exploratory phylogenetic trees

**DOI:** 10.1186/s13104-018-3268-y

**Published:** 2018-03-05

**Authors:** Thomas Sauvage, Sophie Plouviez, William E. Schmidt, Suzanne Fredericq

**Affiliations:** 1 Department of Biology, University of Louisiana at Lafayette, 410 E. Saint Mary Boulevard, Lafayette, LA 70503 USA; 2 Smithsonian Marine Station, 701 Seaway Drive, Fort Pierce, FL 34949 USA

**Keywords:** Barcoding, Biodiversity, Clone, Contaminant, Cryptic, Environmental, FigTree, Forensic, Metabarcoding, OTU, Phylogeny, Systematics

## Abstract

**Objective:**

The body of DNA sequence data lacking taxonomically informative sequence headers is rapidly growing in user and public databases (e.g. sequences lacking identification and contaminants). In the context of systematics studies, sorting such sequence data for taxonomic curation and/or molecular diversity characterization (e.g. crypticism) often requires the building of exploratory phylogenetic trees with reference taxa. The subsequent step of segregating DNA sequences of interest based on observed topological relationships can represent a challenging task, especially for large datasets.

**Results:**

We have written TREE2FASTA, a Perl script that enables and expedites the sorting of FASTA-formatted sequence data from exploratory phylogenetic trees. TREE2FASTA takes advantage of the interactive, rapid point-and-click color selection and/or annotations of tree leaves in the popular Java tree-viewer FigTree to segregate groups of FASTA sequences of interest to separate files. TREE2FASTA allows for both simple and nested segregation designs to facilitate the simultaneous preparation of multiple data sets that may overlap in sequence content.

**Electronic supplementary material:**

The online version of this article (10.1186/s13104-018-3268-y) contains supplementary material, which is available to authorized users.

## Introduction

A classic workflow in DNA-based systematics studies [1] consists in building exploratory trees to visualize topological relationships of novel sequences within a larger framework of reference taxa. This allows for the molecular identification of uncurated sequences, the discovery of molecular crypticism [2], as well as choosing relevant ingroup/outgroup taxa [3] (i.e. those to be segregated among the pool of available FASTA sequences for focused systematics studies). Systematists may also need to segregate groups of FASTA sequences to examine sequence attributes across different clades, such as comparing GC content, examine sequence motifs or divergence. Currently, efficiently mining FASTA sequences of interest from tree topologies can represent a difficult task since tree-viewing relies on a Newick string [4] that does not contain DNA information, the latter being enclosed in the original FASTA file used for tree-building. Thus, to relate DNA strings to tip labels (i.e. sequence names), one usually needs to script in programming language such as R, e.g. relying on the package Ape [5] with function ‘drop.tip’ or ‘extract.clade’ to create object lists of sequence names to match to DNA sequences. While this may facilitate part of the process, rapidly selecting numerous clades or tips interactively in the R interface may not be as fluid as in a dedicated tree-viewer such as the popular Java program FigTree [6]. For researchers with limited scripting skills, the process requires to manually edit FASTA files via copy/paste (or delete) in a text editor for wanted (or unwanted) sequences. Others may type extensive lists of observed tip labels (i.e. sequence names) that can be used to parse FASTA files with dedicated scripts available from the community, or with matching functions of the Galaxy tool shed [7], as well as with command line tools such as samtools [8] or blastdbcmd from the NCBI Blast+ package [9]. Overall, although some of the above practices may be feasible for small datasets (e.g. typing lists), they may rapidly become unpractical for researchers who are faced with large data sets (100 to 1000+ sequences to be sorted). Here, to offer a rapid and interactive solution to sequence selection from exploratory phylogenies, we devised a Perl script named TREE2FASTA that allows the batch extraction of FASTA sequences via color and/or annotation of tips/clades of interest in FigTree. We illustrate an example use of this Perl script to rapidly sort unidentified chloroplast 16S rDNA sequences belonging to the red seaweeds from the public NCBI Genbank^®^ repository. We also document TREE2FASTA’s execution speed on two 1000+ sequence trees built from reference databases used in phototroph metabarcoding.

## Main text

### General implementation

**Fig. 1:**
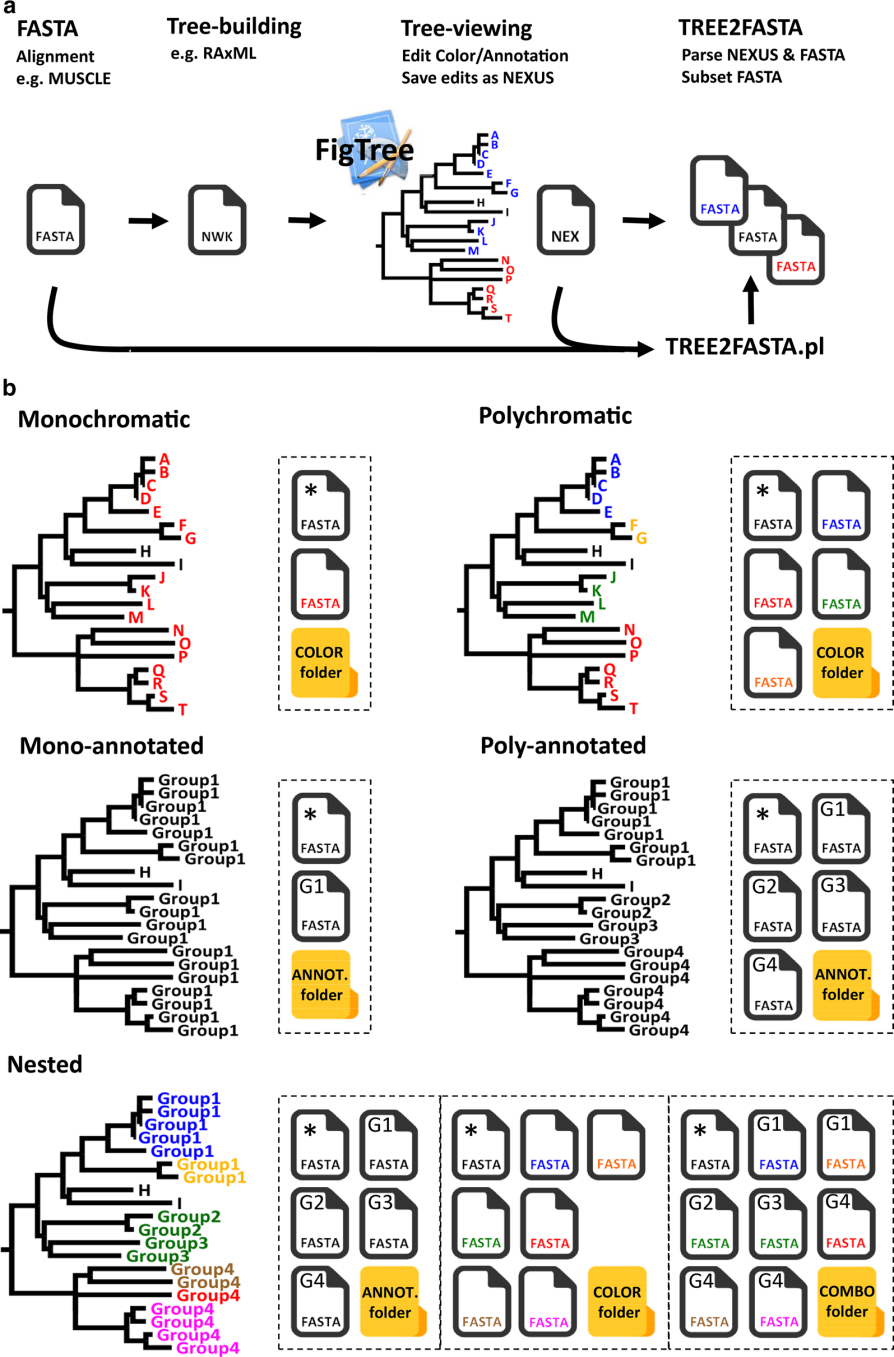
Simulated phylogeny displaying taxa named ‘A’ to ‘T’. **a** Basic workflow for FASTA sequence extraction with TREE2FASTA. An exploratory tree is built following multiple-alignment of FASTA data. The Newick tree string (NWK) is visualized and edited in the tree-viewer FigTree and saved as a NEXUS file (NEX). TREE2FASTA uses the FASTA alignment and the NEXUS file (NEX) to produce subsetted FASTA files according to user selection scheme (here color). **b** Example of possible color and/or annotation selection schemes in FigTree for TREE2FASTA sequence extraction. The FASTA icon marked with an asterisk ‘*’ contains FASTA sequences for taxa H and I lacking color selection (i.e. achromatic) or lacking annotation. For figure clarity annotation ‘Group1’ to ‘Group4’ are reported G1 to G4 within FASTA file icons. FASTA files output to different folders are delimited by dashed boxes

A preliminary requirement to using TREE2FASTA is to produce an exploratory phylogeny in a tree-building program relying on a FASTA sequence alignment (e.g. in RAxML [10]). Such program will output a Newick string (‘.nwk’ or ‘.tre’ file) directly readable by most tree-viewers for visualization and editing (Newick strings encode taxa relationships in a simple nested parentheses format embracing sequence labels [4]). Here, we specifically chose the tree-viewer FigTree for its intuitive and interactive interface allowing rapid coloring/annotation in a few mouse clicks, and for conveniently exporting edited information within NEXUS files [11]. TREE2FASTA exclusively parses the taxa block of FigTree’s NEXUS file, which contains sequence labels and edited information (see Additional file 1), in order to match sequence labels in the original FASTA file (containing the DNA sequences) for batch extraction of the sequences of interest in subsetted FASTA files (Fig. 1a). Sequence extraction follows the selection scheme edited on the tree by the user.

### Input and output

TREE2FASTA formats input files’ end of lines to line feed (LF) character and thus can be run on Linux, Mac and Windows systems with files generated on any of (and across) these platforms. The user provides an interleaved or sequential FASTA file (i.e. DNA in multi-line or single-line, respectively) and a NEXUS tree file exclusively edited from FigTree. While running, TREE2FASTA will check for the concordance of FASTA sequence identifiers with tree labels, as well as for duplicates within each of these files. TREE2FASTA will issue warnings and print duplicate or missing sequence labels to help with any troubleshooting. However, in such instance, TREE2FASTA will still run forward and extract existing matches for flexibility. Subsetted FASTA files are placed in dedicated folders (‘ANNOT’, ‘COLOR’, ‘COMBO’) and each FASTA files is named according to the annotation or color edited on the tree (as hexadecimal (HEX) codes according to the encoding of individual colors in FigTree’s NEXUS file, Additional file 1) or as an annotation_color combination. TREE2FASTA fully deconstructs the edited tree design (each color or annotation component and their combination) so users can pick the FASTA files most relevant to their splitting goals.

### Selection schemes

For the purpose of illustration, we use a small simulated phylogeny with taxa denoted A to T that we color and/or annotate as Group1 to Group4. Five main examples are displayed, single and multiple colors, single and multiple annotations, and the nested combination of colors and annotations (Fig. 1b). For simplicity here, we depicted TREE2FASTA outputs as FASTA icons with colors matching the tips in the tree (rather than with HEX codes, see section above) and with shorter annotations (in top left corner of FASTA icon, e.g. ‘G1’ instead of ‘Group1’). Note that when using annotation in FigTree, this program masks the original tip label on the tree but keeps track of it internally.

Using a single color or annotation (i.e. monochromatic or mono-annotated) will result in the output of two files, one containing all edited sequences (FASTA icon red or FASTA icon ‘G1’ for Group1, respectively), and the second, the remaining unselected sequences (i.e. taxa H and I in the FASTA icon denoted with an asterisk; such files are named ‘NOCOLOR.fas’ or ‘NONAME.fas’ in TREE2FASTA computer output). Likewise, using multiple colors or annotations (i.e. polychromatic or poly-annotated selection) will result in the output of multiple files for each of the edited colors or annotations. As above, unselected taxa are segregated in their own FASTA file.

For more complex selection of taxa, colors and annotations may be combined in a nested manner. Nesting is most useful for the simultaneous extraction of the same FASTA sequence (or group of sequences) to separate files (e.g. common sequences found across a higher and lower taxonomic level). Nesting schemes may be designed by color or by annotation (i.e. a colored clade with multiple annotations, or an annotated clade with multiple colors). For instance, in the nested example (Fig. 1b), Group4 shows nesting by color (same annotation, multiple colors) while Group2 and Group3 are nested by annotation (multiple annotation, same color). All FASTA sequences for Group4 (nested by color) will be found in the ANNOT. folder (FASTA icon ‘G4’) while FASTA sequences for its subclades (edited for different colors) will be found in the COLOR folder (FASTA icon with brown, red and pink files). Similarly, all FASTA sequences for Group2 and Group3 (nested by annotation) can be found in the COLOR folder (green file), while subsetted sequences for each of the two groups can be found in the ANNOT. folder (FASTA icon ‘G2’ and ‘G3’). The COMBO folder contains fully subsetted FASTA files corresponding to all nested groups according to the combined color/annotation selection scheme; the next section presents a real-world example of its utility.

### Example of public data mining

**Fig. 2:**
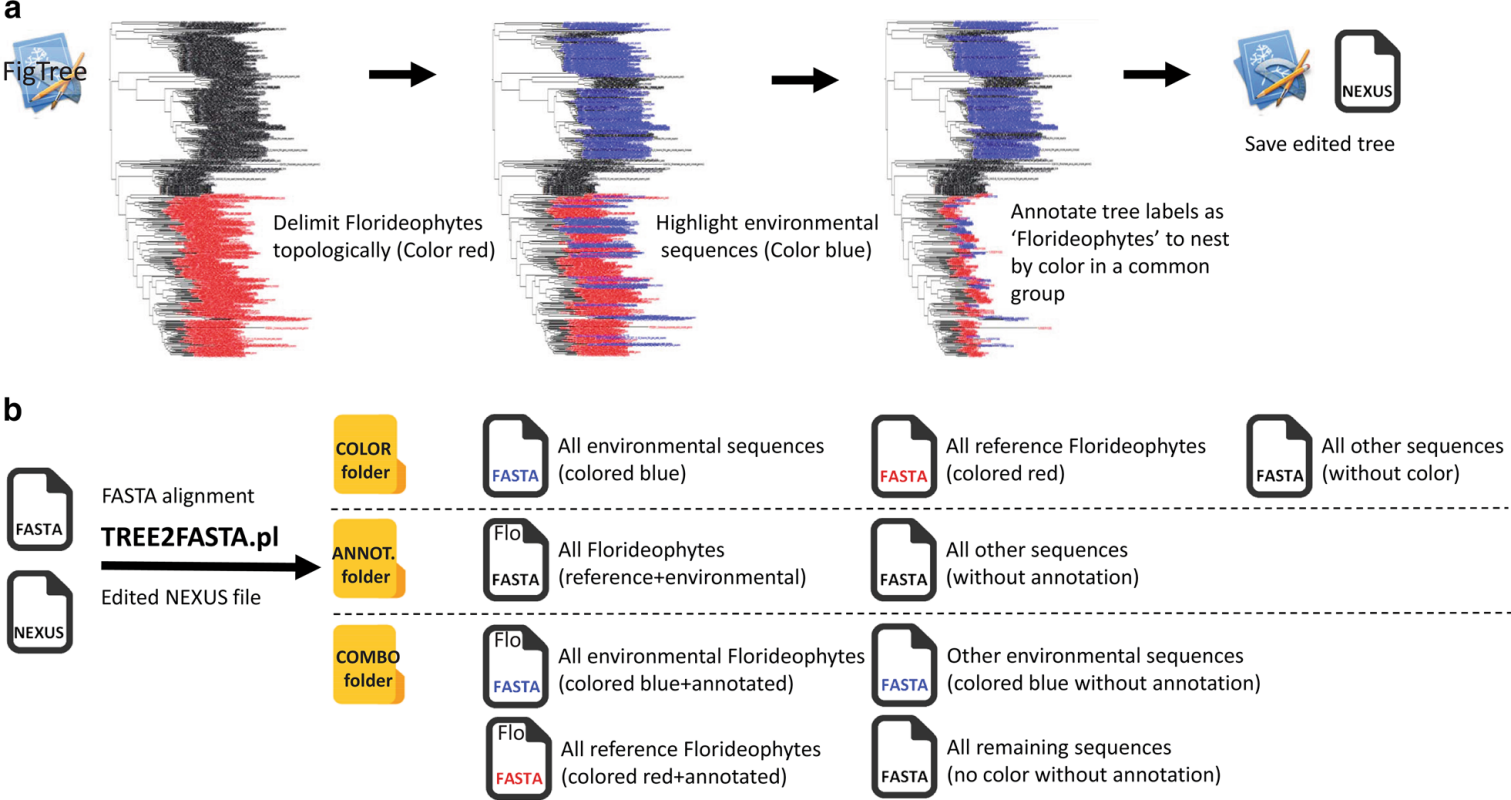
Sorting Genbank 16S rDNA for red seaweeds with TREE2FASTA. **a** Successive edits done in FigTree to establish an annotated design nested by color for reference and environmental red seaweeds (Florideophytes). **b** Folders and subsetted FASTA files output by TREE2FASTA for downstream analyses (folder content separated by dashed lines). For figure clarity, the Florideophyceae annotation was abbreviated to ‘Flo’ within FASTA file icons. The tree was produced with the 500 closest matches to *Taenioma perpusillum* (MF101452) on Genbank^®^

Public databases such as Genbank^®^ abound with unidentified/misidentified environmental sequence data (clones, OTUs) whose headers lack informative taxonomy (e.g. “Uncultured organism”). These cannot always be parsed easily from the mass of available data and often necessitate BLASTn search followed by tree-building for their identification based on topological relationships. In this example, we would like to retrieve a comprehensive FASTA data set of chloroplast 16S rDNA for phylogenetic reconstruction of red seaweeds (class Florideophyceae, phylum Rhodophyta). We are interested in producing several datasets simultaneously, including segregating all reference sequences with implicit taxonomy, all environmental sequences without taxonomy (e.g. to facilitate header curation for databasing), and all red seaweeds. To do so, we downloaded the 500 closest matches found on Genbank^®^ (via BLASTn) to the 16S sequence of a red seaweed. Following multiple-alignment of the sequences in MUSCLE (2 iterations) [12], we built an exploratory maximum likelihood (ML) tree with RAxML (keeping the best tree out of 10 restart searches with the rapid hill-climbing algorithm; although fewer restarts may be sufficient, i.e. 1–5) (see Additional file 2 for example command lines to these programs). The resulting Newick string was imported in the tree-viewer FigTree (v1.4.2, [6]) and topological boundaries of the Florideophyceae delimited with the color red in one mouse click (Fig. 2a, see Additional file 3 for our tutorial on FigTree). We then used FigTree’s search field with the word “uncultured” to highlight all environmental sequences and colored them in one mouse click in blue. Finally, we moused over the entire clade of the red seaweeds to rapidly annotate all tree tip labels at once as “Florideophyceae”, thus creating a nesting design by linking red and blue tips internally under this annotation. The edited tree was saved to disk from FigTree as a NEXUS file and used as input to TREE2FASTA along with the above FASTA file alignment. Several subsetted FASTA files were produced. These correspond to the full deconstruction of the tree selection design (color, annotation and combination). Among the file outputs, the most relevant to the initial splitting goals (i.e. finding all red seaweeds and segregating references from environmental representatives), is the file found in the ANNOT. folder containing all Florideophytes (annotation shortened as ‘Flo’ in the FASTA icons, Fig. 2b), and files in the COMBO folder, containing the reference Florideophytes (red FASTA file icon with ‘Flo’) and environmental Florideophytes (blue FASTA file icon with ‘Flo’) in separate files (Fig. 2b). A detailed view of the tree (Additional file 4) shows that many of the environmental sequences representing red seaweeds are misidentified as “uncultured bacterium” or “uncultured cyanobacterium” or poorly identified as “uncultured organism” or “uncultured phototrophic eukaryote”, and are thus easily overlooked in public databases in spite of their value for molecular systematics. In this example, TREE2FASTA provides a simple workflow for their facilitated recovery.

### Execution speed

To document TREE2FASTA’s execution speed, we downloaded two FASTA sequence reference databases of use in phototroph metabarcoding, *tuf*A [13, 14] and the 16S rDNA PhytoREF [15] (Table 1). We produced exploratory ML trees (as above) and edited 10 phyletic clades in FigTree with color and with color + annotation (Additional file 5). Using an iMac (3.5 GHz Intel Core i7 processor with 4 physical cores and 32 GB DDR3 RAM memory), we measured fast execution speed of our script with elapsed wall-clock time inferior to < 15 s for the largest data sets (Table 1). Note that in the above example, browsing and editing the > 4000 PhytoREF sequence tree in FigTree remained extremely fluid.

**Table 1:** Elapsed time for TREE2FASTA execution on 1000+ sequence FASTA datasets

Database (edited scheme)	Sequences	Length (bp)	Wall-clock	CPU
*tuf*A (color)	1957	483	1.055	1.025
*tuf*A (color + annotation)	1957	483	1.056	1.040
16S PhytoREF (color)	4191	3379	12.973	12.646
16S PhytoREF (color + annotation)	4191	3379	13.164	12.871

Time reported as wall-clock (= ‘real’) and CPU (‘user’ + ‘sys’). See text for computing system specifications. Length refers to the sequence multiple-alignment length [in base pair (bp)]

### Conclusions

TREE2FASTA allows for the interactive, flexible and rapid sorting of FASTA sequences from clades of interest with minimal user efforts via the popular tree-viewer FigTree. In its simplest application, e.g. monochromatic, TREE2FASTA can be used to rapidly select ingroup/outgroup taxa following exploratory analyses, a nearly ubiquitous task in phylogenetics leading toward publication-ready trees. Considering that TREE2FASTA allows the tracking of DNA sequences based on topological relationships, the script is particularly suitable to the sorting of FASTA-formatted data containing misidentified (and contaminants) or unidentified/environmental sequences (barcodes and metabarcodes), such as those generated in molecular ecology, forensics and biodiversity exploration projects.

## Limitations

- We recommend using the RAxML tree-building program. RAxML preserves the sequence header format from FASTA input to Newick string output. TREE2FASTA will print any discordant sequence headers between files for troubleshooting.
- Because user-friendly color names (e.g. white, red) are neither available nor definable for the 16,777,216 existing HEX codes, TREE2FASTA reports color FASTA files names as HEX codes (as in FigTree’s NEXUS files). Users may open the “summary_by_color” file to rapidly relate their HEX color file name to extracted sequences.

## Additional files

**Additional file 1.** FigTree Tutorial and command line usage for TREE2FASTA.

**Additional file 2.** Details of color and annotation information saved within the TAXA block of FigTree’s NEXUS file.

**Additional file 3.** Example command line for multiple alignment in MUSCLE and maximum likelihood estimation RAXML to rapidly produce exploratory tree.

**Additional file 4.** Details of the Florideophyceae clade shown in Fig. 2a.

**Additional file 5.** Illustration of the edited exploratory tree used to measure TREE2FASTA execution speed reported in Table 1. Both trees were edited for color and/or annotation in FigTree for 10 phyla of the *tuf*A and 16S PhytoREF database. Color details: Chlorarachniophyta (CYAN), Chlorophyta (GREEN), Euglenozoa (BLUE), Cryptophyta (BROWN), Rhodophyta (RED), Glaucophyta (MAGENTA), Ochrophyta (ORANGE), Haptophyta (YELLOW), Cyanophyta (BLACK), Streptophyta (PURPLE). Note that smaller phylum clades, albeit present in both trees are not easily visible among other colors (e.g. Cryptophyta, Euglenozoa). Cyanophyta is absent from the PhytoREF. Streptophyta is absent for the *tuf*A database.

**Additional file 6.** Example tree file for the nested example shown in Fig. 1.

**Additional file 7.** Example FASTA file for the nested example shown in Fig. 1.

## Abbreviations

HEX: hexadecimal
LF: line feed
ML: maximum likelihood
NWK: Newick
NEX: Nexus
