# Supplementary material for: TREE2FASTA: a flexible Perl script for batch extraction of FASTA sequences from exploratory phylogenetic trees
Source: BMC Res Notes. 2018 Mar 5;11:164. doi: 10.1186/s13104-018-3268-y (PMC5838971; doi:10.1186/s13104-018-3268-y)
Supplement: Supplementary file 1 — Additional file 1. FigTree Tutorial and command line usage for TREE2FASTA. [file 13104_2018_3268_MOESM1_ESM.pdf]

```
#NEXUS
begin taxa;
	dimensions ntax=20;
	taxlabels
	A[&!color=#0004ff,!name="Group1"]
	B[&!color=#0004ff,!name="Group1"]
	C[&!color=#0004ff,!name="Group1"]
	D[&!color=#0004ff,!name="Group1"]
	E[&!color=#0004ff,!name="Group1"]
	F[&!color=#ffaf24,!name="Group1"]
	G[&!color=#ffaf24,!name="Group1"]
	H
	I
	J[&!color=#1bbd13,!name="Group2"]
	K[&!color=#1bbd13,!name="Group2"]
	L[&!color=#1bbd13,!name="Group3"]
	M[&!color=#1bbd13,!name="Group3"]
	N[&!color=#996633,!name="Group4"]
	O[&!color=#996633,!name="Group4"]
	P[&!color=#ff0008,!name="Group4"]
	Q[&!color=#800080,!name="Group4"]
	R[&!color=#800080,!name="Group4"]
	S[&!color=#800080,!name="Group4"]
	T[&!color=#800080,!name="Group4"]
;
end;
```
