# Supplementary material for: TREE2FASTA: a flexible Perl script for batch extraction of FASTA sequences from exploratory phylogenetic trees
Source: BMC Res Notes. 2018 Mar 5;11:164. doi: 10.1186/s13104-018-3268-y (PMC5838971; doi:10.1186/s13104-018-3268-y)
Supplement: Supplementary file 2 — Additional file 2. Details of color and annotation information saved within the TAXA block of FigTree’s NEXUS file. [file 13104_2018_3268_MOESM2_ESM.pdf]

Building multiple alignments and exploratory trees with MUSCLE and RAxML

Example command line for stand-alone installation of MUSCLE and RAXML

#MUSCLE commands for 2 iterations

```
muscle -in tufA.fas -seqtype auto -maxiters 2 -out tufA_aln.fasta
```

#RAXML for 10 restarts with 4 threads

```
raxml -s tufA_aln.fasta -n tufA -m GTRGAMMA -p 5463789 -T 4 -N 10
```
