# Supplementary material for: TREE2FASTA: a flexible Perl script for batch extraction of FASTA sequences from exploratory phylogenetic trees
Source: BMC Res Notes. 2018 Mar 5;11:164. doi: 10.1186/s13104-018-3268-y (PMC5838971; doi:10.1186/s13104-018-3268-y)
Supplement: Supplementary file 3 — Additional file 3. Example command line for multiple alignment in MUSCLE and maximum likelihood estimation RAXML to rapidly produce exploratory tree. [file 13104_2018_3268_MOESM3_ESM.pdf]

FigTree Tutorial and TREE2FASTA Usage

#Preliminary notes:

It is important to quit FigTree prior to reloading a tree.
FigTree will sometimes keep track of colors or annotations for a given sequence label internally and thus display prior edits on screen even if not asked to do so.

To zoom in large trees, use the '<u>expansion</u>' ruler (see image below on the left) rather than the '<u>zoom</u>' ruler.

Annotations will replace the original tree labels on screen but FigTree keeps track of the original taxa names internally. Thus, If you will do color + annotation, it is better to color first.

To save your edited tree as NEXUS file, use the '<u>Save</u>' button in the drop down 'File' menu.
DO NOT USE the '<u>Export Trees…</u>' function which does not print a TAXA block with the edited information.

#Editing trees in FigTree v.1.4.3 for TREE2FASTA

Open the tree file in FigTree
Locate the 'TAXA' tabs and click on it
Locate the 'Colour' tab, 'Annotate' tab and the search field

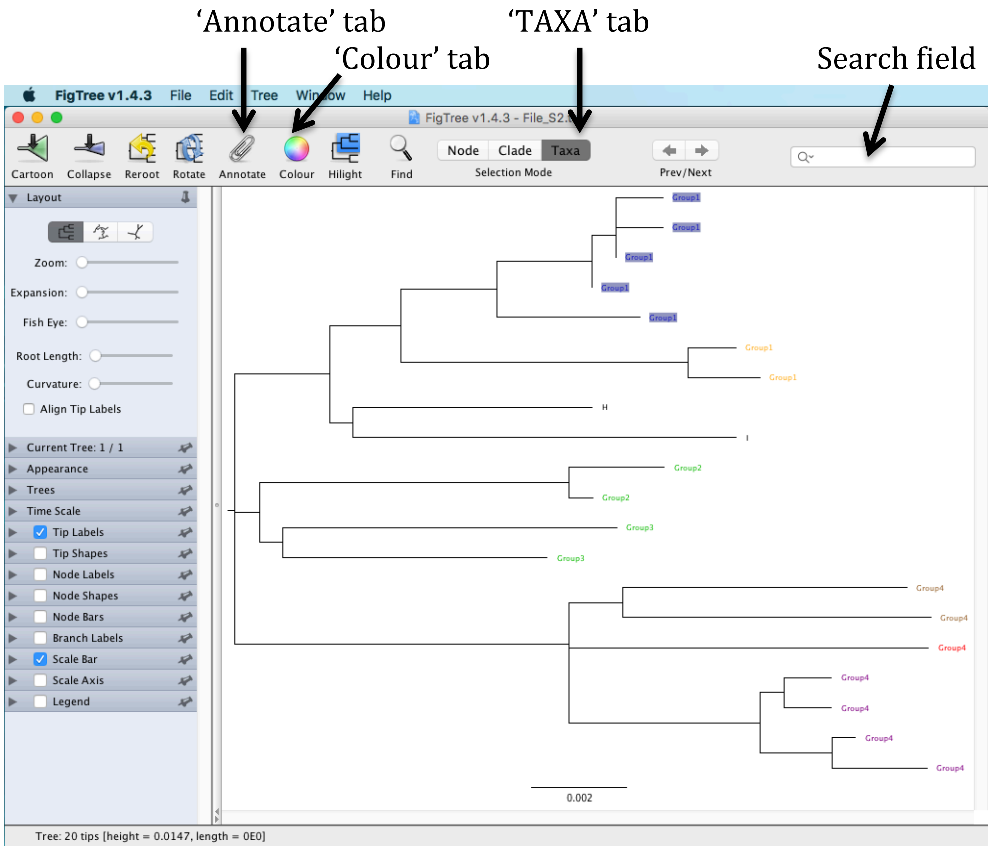

1- Select the group of sequences of interest

if the group is **monophyletic:**

Click on branch/node uniting the clade; all taxa will be selected at once.

Click on the 'Colour' and select the color you want to assign to the group, Click 'OK'

and/or

Click on the 'Annotate' tab, type in the desired annotation in the 'value' field, Click 'OK'

if the group is **polyphyletic**, repeat as above for each group/branch according to the selection scheme desired

Notes:

Groups and branches may also be selected by mouse hover, or via the search field on the top right corner of the main banner.

Annotations will replace the original tree labels on screen but FigTree keeps track of the original taxa names internally.

Taxa lacking color selection (i.e. achromatic) or annotation will be considered as a separate group by TREE2FASTA. Thus not all clades/taxa need to be colored/annotated. Such groups will be output by TREE2FASTA in fasta files 'NOCOLOR.fas' or 'NONAME.fas'.

2- Save as NEXUS file

Use FigTree's main menu bar 'File', then 'Save' or 'Save as'

Note:

DO NOT USE the 'Export Trees…' function to save the tree!
TREE2FASTA will not be able to parse the file produced.

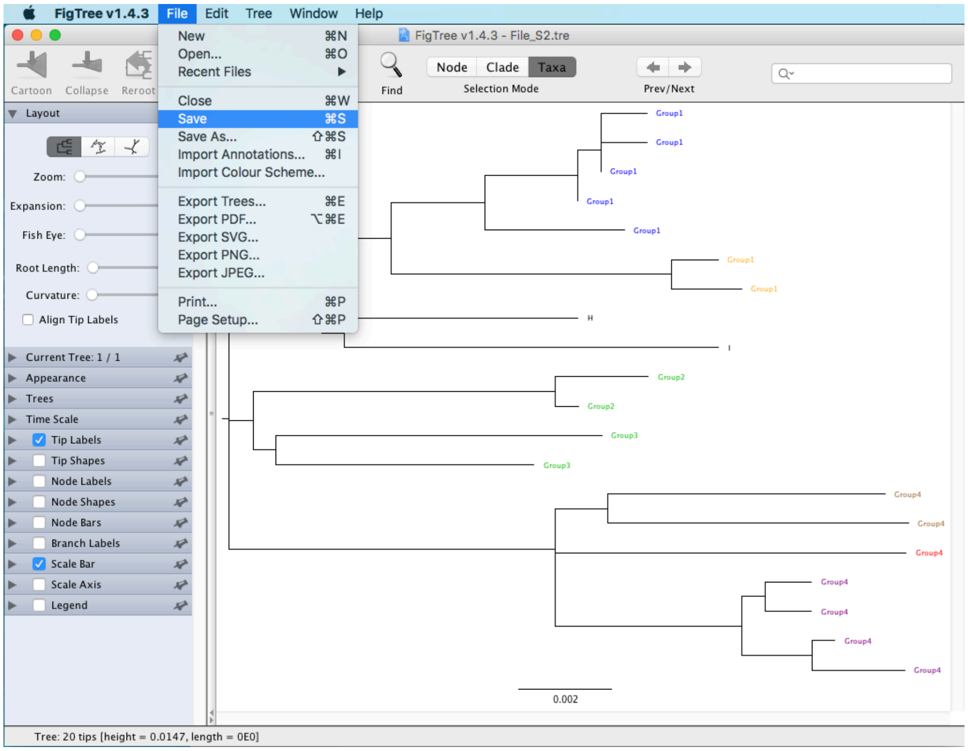

TREE2FASTA usage

1 - Navigate with terminal to the desired working directory

cd   path_to_working_directory

Place TREE2FASTA.pl, the edited tree file (FigTree's NEXUS) and the FASTA file in this working directory (or indicate the paths to the location of the script and files in the command line).

2- Run TREE2FASTA as follows[1]:

perl   TREE2FASTA.pl   tre_file_name   fasta_file_name

Example command line with supplementary files provided

perl   TREE2FASTA.pl   example_tree.tre   example_fasta.fas

To see command line usage for TREE2FASTA, type:

perl   TREE2FASTA.pl

---

[1] In case the tree file and FASTA file are not provided in the correct order, TREE2FASTA will stop and ask you to provide files in the correct order. The Tree file should be first and the FASTA file second in the command line – as a mnemonic, just remember the name of the script, TREE-TO-FASTA.
