# Supplementary figures and images for: TREE2FASTA: a flexible Perl script for batch extraction of FASTA sequences from exploratory phylogenetic trees
Source: BMC Res Notes. 2018 Mar 5;11:164. doi: 10.1186/s13104-018-3268-y (PMC5838971; doi:10.1186/s13104-018-3268-y)

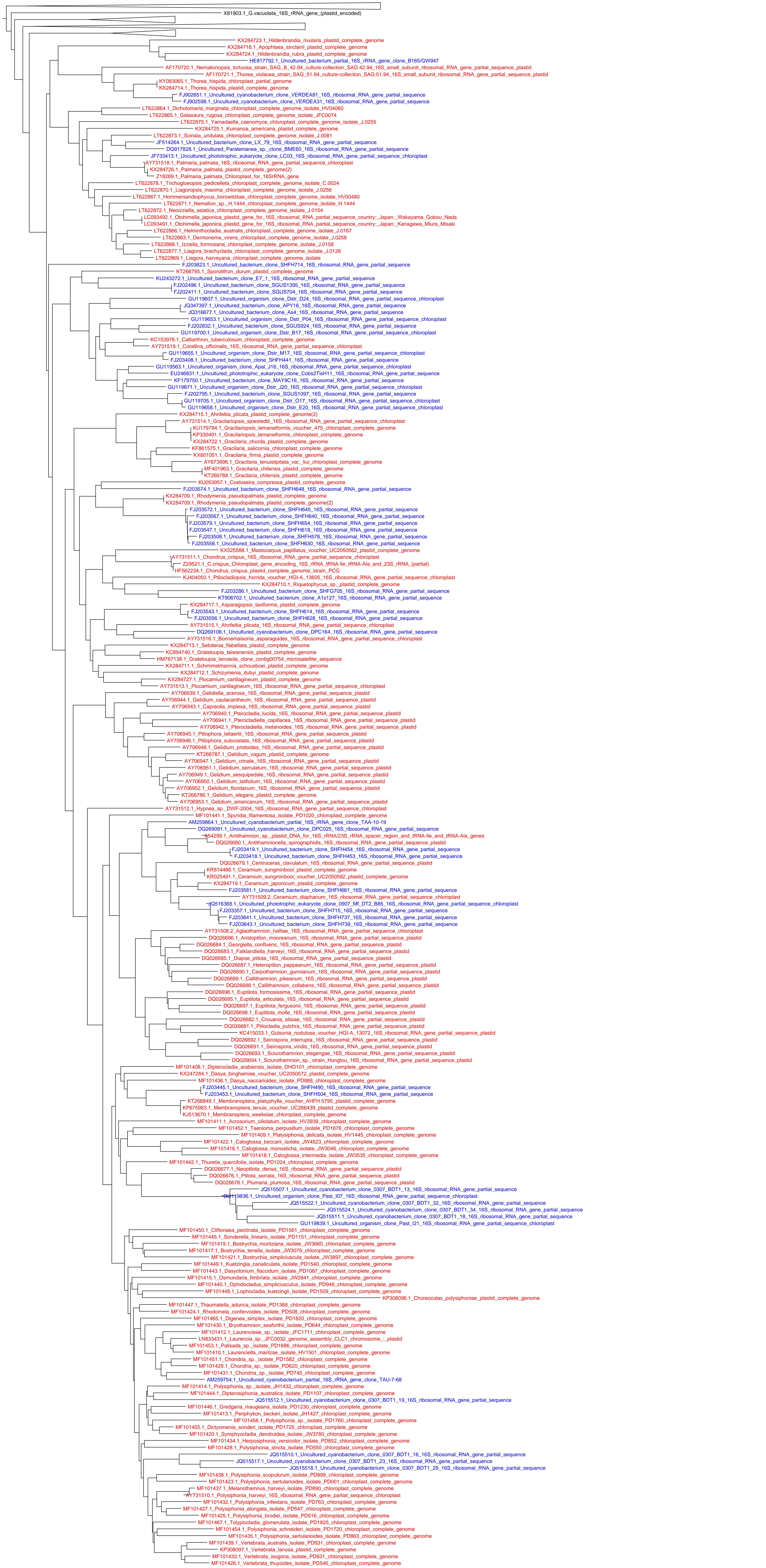

Supplement: Supplementary file 4 — Additional file 4. Details of the Florideophyceae clade shown in Fig. 2a. [file 13104_2018_3268_MOESM4_ESM.pdf]
