# Supplementary material for: TREE2FASTA: a flexible Perl script for batch extraction of FASTA sequences from exploratory phylogenetic trees
Source: BMC Res Notes. 2018 Mar 5;11:164. doi: 10.1186/s13104-018-3268-y (PMC5838971; doi:10.1186/s13104-018-3268-y)
Supplement: Supplementary file 5 — Additional file 5. Illustration of the edited exploratory tree used to measure TREE2FASTA execution speed reported in Table 1. Both trees were edited for color and/or annotation in FigTree for 10 phyla of the tufA and 16S PhytoREF database. Color details: Chlorarachniophyta (CYAN), Chlorophyta (GREEN), Euglenozoa (BLUE), Cryptophyta (BROWN), Rhodophyta (RED), Glaucophyta (MAGENTA), Ochrophyta (ORANGE), Haptophyta (YELLOW), Cyanophyta (BLACK), Streptophyta (PURPLE). Note that smaller phylum clades, albeit present in both trees are not easily visible among other colors (e.g. Cryptophyta, Euglenozoa). Cyanophyta is absent from the PhytoREF. Streptophyta is absent for the tufA database. [file 13104_2018_3268_MOESM5_ESM.pdf]

We downloaded two databases that differ in number of sequences and character length. The *tuf*A dataset [13, 14] was >1900 sequences with a length of 480 bp while the 16S rDNA PhytoREF [15] was >4000 sequences with a length of 3380 bp (about 2000 sequences that were short, with introns, or of lower quality, i.e. with IUAPC characters, were removed). We colored and colored+annotated 10 phyletic clades for each datasets (Chlorarachniophyta - CYAN, Chlorophyta - GREEN, Euglenozoa - BLUE, Cryptophyta - BROWN , Rhodophyta - RED, Glaucophyta - MAGENTA, Ochrophyta - ORANGE, Haptophyta - YELLOW, Cyanophyta - BLACK, Streptophyta/Charophyta – PURPLE).

# Color

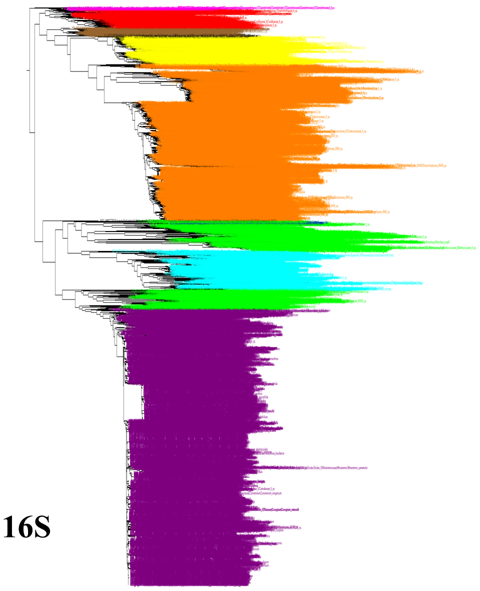

16S

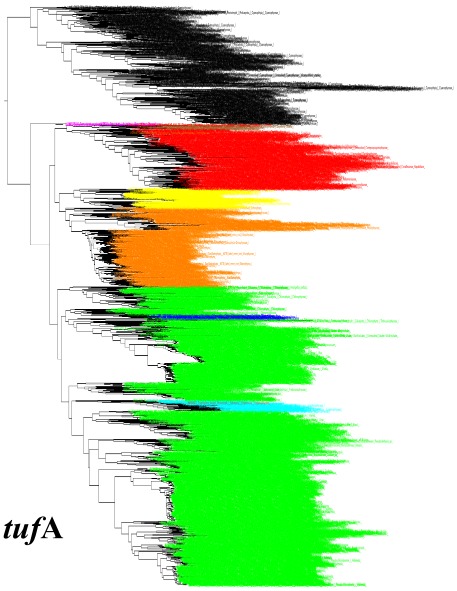

***tuf*A**

# Color + Annotation

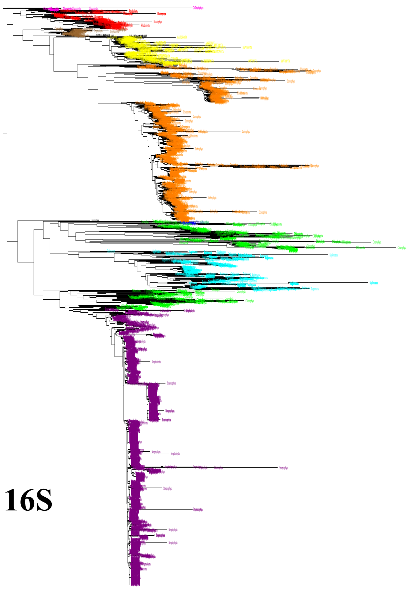

16S

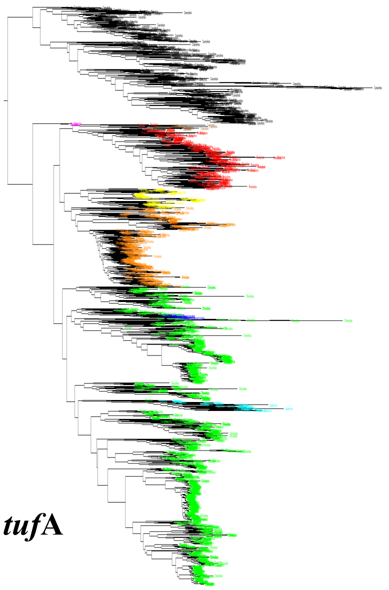

***tuf*A**
